# Electrophysiological Evidence for Immature Processing Capacity and Filtering in Visuospatial Working Memory in Adolescents

**DOI:** 10.1371/journal.pone.0042262

**Published:** 2012-08-22

**Authors:** Marjolein Spronk, Edward K. Vogel, Lisa M. Jonkman

**Affiliations:** 1 Department of Cognitive Neuroscience, Maastricht University, Maastricht, The Netherlands; 2 Department of Psychology, University of Oregon, Eugene, Oregon, United States of America; University of Potsdam, Germany

## Abstract

The present study investigated the development of visuospatial working memory (VSWM) capacity and the efficiency of filtering in VSWM in adolescence. To this end, a group of IQ-matched adults and adolescents performed a VSWM change detection task with manipulations of WM-load and distraction, while performance and electrophysiological contralateral delay activity (CDA) were measured. The CDA is a lateralized ERP marker of the number of targets and distracters that are selectively encoded/maintained in WM from one hemifield of the memory display. Significantly lower VSWM-capacity (Cowan's *K*) was found in adolescents than adults, and adolescents' WM performance (in terms of accuracy and speed) also suffered more from the presence of distracters. Distracter-related CDA responses were partly indicative of higher distracter encoding/maintenance in WM in adolescents and were positively correlated with performance measures of distracter interference. This correlation suggests that the higher interference of distracters on WM performance in adolescents was caused by an inability to block distracters from processing and maintenance in WM. The lower visuospatial WM-capacity (*K*) in adolescents in the high load (3 items) condition was accompanied by a trend (*p*<.10) towards higher CDA amplitudes in adolescents than adults, whereas CDA amplitudes in the low load (1 item) condition were comparable between adolescents and adults. These findings point to immaturity of frontal-parietal WM-attention networks that support visuospatial WM processing in adolescence.

## Introduction

Working memory (WM), our system for storage and maintenance of information for short periods of time, plays a crucial role in the development of cognitive abilities. Especially WM-capacity is an important factor for the development of academic skills such as reading and mathematics [1], [2] and fluid intelligence [3], [4].

The limits of WM-capacity have been extensively researched, leading to the assumption that adults can only maintain a maximum of three to four items simultaneously in WM [5], [6], [7]. However, recent work has shown that the capacity of WM does not only depend on how many items can be stored in short-term memory, but also on the efficiency with which items are stored [8], [9], [10]. The latter means that only items that are relevant to current task goals should be selected for access to and maintenance in WM. Selective attention, mediated by fronto-striatal-parietal networks, is thought to play an important role in this regulation of access to WM [8], [10], [11], [12]. Recent studies have shown that low WM-capacity can indeed be caused by inefficient filtering of information that enters WM for maintenance [8], [11], [12], [13], [14], [15].

Developmental studies have consistently shown that mature WM-capacity is only reached during late childhood or adolescence. Whereas some studies report mature visuospatial working memory (VSWM) capacity around 10–12 years of age [16], [17], other studies report that mature WM-capacity is not reached before the age of 16 [18], [19], [20]. Such developmental differences seem to depend on the level of executive control processes that are needed to perform a specific WM task. As mentioned above, one such important executive process is filtering efficiency, that is, the efficiency with which the individual is capable of excluding irrelevant information to get access to, or interfere with the current contents of WM. Schleepen and Jonkman [21] showed particularly late development of non-spatial WM-capacity into adolescence in task conditions requiring simultaneous maintenance, updating and suppression of irrelevant information.

This late development of WM-capacity in tasks demanding high attentional control has been attributed to the protracted development of fronto-striatal-parietal brain networks that are known to be involved in regulating access to WM [12], [22], [23], [24]. In healthy adults, activations of parietal structures like the intraparietal sulcus (IPS) during VSWM tasks have been found to increase with WM-load, leveling off at maximum capacity and are hence thought to be associated with storage capacity [7], [25], [26]. The dorsolateral prefrontal cortex (DLPFC) on the other hand is thought to play an important role in attentional control over which information should be maintained and rehearsed in WM in the retention interval of a task [27]. Increased activity in DLPFC during WM tasks has been observed especially under high WM-load conditions [28], [29], suggesting frontal areas are essential in keeping performance levels high through cognitive control. Moreover, Edin et al. [30] have found that DLPFC might also be able to boost parietal areas (IPS) to achieve higher WM-capacity during a visuospatial WM task. Bunge and Wright [22] reviewed the developmental functional imaging literature in the field of spatial attention and WM: They reported that better performance on WM tasks across age, especially when these tasks require more cognitive control, coincides with increased recruitment of frontal and parietal areas, like DLPFC and IPS.

In most of the developmental studies mentioned above, conclusions about developmental differences in storage capacity were derived from performance measures such as differences in recognition or change detection accuracy in tasks in which test displays have to be compared to what has been earlier stored in WM. Before arriving to a response in such tasks subjects pass through several processing stages, namely encoding, maintenance and a comparison/decision process. Thus, there are several possible origins from where developmental differences in VSWM performance between adults and adolescents can arise. Behavioral studies cannot exclude that other processes than encoding or maintenance are responsible for performance differences in WM tasks. Therefore, in the current study a lateralized event-related brain potential (ERP) marker above parietal cortex that is indicative of the number of targets (or distracters) that are selectively encoded and maintained from one hemifield of the memory display will be also measured.

The contralateral delay activity (CDA) was first reported by Vogel and Machizawa [31]. These authors found that in healthy adults, the CDA increased in amplitude with the number of items maintained in WM and they also reported a correlation between CDA amplitude and a behavioral measure of VSWM-capacity [5]. Since this first report, the CDA has been used as a neural correlate of the number of items encoded and maintained in WM in a number of other studies including healthy adults, elderly, or patient groups [32], [33], [34], [35]. In another study by Vogel et al. [8] it was shown that the CDA could also be used as a measure of filtering efficiency since it also increased in amplitude when irrelevant items were encoded for storage in WM, despite instructions. This was shown in one experiment where subjects had to memorize the orientation of either two or four red items (the targets) for a later memory test. In a third condition two target items were accompanied by two distracter (e.g. blue-colored) items (T2D2; 2 targets, 2 distracters) that should not be stored in WM. It was expected that subjects with inefficient filtering abilities would also store these two distracter items in WM, having a total storage load of four items. Taken that the CDA amplitude varies with the number of stored items, this should be reflected by overlapping CDA's in the T4D0 and T2D2 conditions. This was indeed what was found, but only in subjects with low-WM-capacity. In subjects with high WM-capacity the T2D2-CDA overlapped with the T2D0-CDA and was smaller than the T4D0-CDA, showing that they had successfully prevented storage of the two distracter items. Thus, in this paradigm, the CDA can be used as a neural correlate of the number of relevant and irrelevant items that are encoded and maintained in memory in the retention interval of the task.

In the present study the change-detection filter task and the ERP-CDA measure were used to investigate the development of VSWM capacity and filtering efficiency in adolescence and enhance insight into to the underlying processes. For this purpose, we compared performance and CDA amplitudes of adolescents and adults performing a VSWM change detection task. To our knowledge, this is the first time that the CDA has been used to investigate the online storage of irrelevant and relevant information in VSWM in adolescence. In the present task three conditions are relevant; a one target, no distracter condition (T1D0), a three target, no distracter condition (T3D0) and a one target, two distracters condition (T1D2). Cowan's *K* was computed from the change-detection task performance data as a behavioral VSWM-capacity measure that estimates each individual's mean number of objects memorized, corrected for guessing [5]. First, lower WM-capacity in adolescents than adults was expected to be reflected by lower *K* scores. Also, in addition to Cowan's *K*, we obtained another measure of WM-capacity -independent from the change detection task- from a Digit Span task [36]. Performance on the Digit Span task has been shown to correlate with performance in other WM tasks that require executive control [37] and was also expected to be lower in adolescents. On the basis of the prior CDA literature [31] we expected that if adults have higher WM storage capacity than adolescents this will be reflected by higher T3D0 CDA amplitudes (relative to T1D0) (e.g. larger CDA increases with load) in adults than adolescents. Based on previous studies larger distracter processing in adolescents would present itself as 1) relatively larger slowing of and/or enhanced errors in change detection performance in the distracter (T1D2) condition than in the no-distracter condition (T1D0) and 2) relatively larger CDA amplitude increases in the T1D2 condition compared to T1D0 and smaller T1D2-T3D0 differences.

## Methods

### Ethics Statement

The present study was approved by the Local Ethical Committee of the Faculty of Psychology and Neuroscience at Maastricht University, and prior to the study a written informed consent was obtained from the children and their caretakers and the adults according to the Declaration of Helsinki (1964). All subjects were paid for their participation in the experiment.

### Subjects

Forty-three subjects participated in the study (23 adolescents and 20 adults), of which five were excluded from the analysis, two in the adolescent group and three in the adult group. One adolescent scored above threshold on the ADHD subscale of the Youth Self Report and Child Behavior Checklist [38], [39] and another adolescent had a hit-percentage below 55% in the T1D0 condition. These subjects were therefore excluded from the analysis. One adult was excluded due to incomplete data collection, a second because hit percentage was too low compared to the other adults (studentized residual >2.5, hit percentage of 59% in T1D0), and a third was excluded to match the groups on IQ. The remaining 21 adolescents (ten boys and eleven girls) were recruited from a school providing vocational education for 12- to 16-year-olds. The 17 adults (eight males and nine females) were recruited via advertisements in local newspapers, and were required to have educational levels comparable to that of the adolescents.

Mean age was 14.8 years (SD 1.4, range 12–16 years) in the Adolescent group and 31.6 years (SD 8.9, range 20–45 years) in the Adult group.

To check for the absence of attention- and/or ADHD behavioral problems, the adolescents themselves filled out the Youth Self Report form [38] and one of their parents filled out the Child Behavior Checklist [39]. Mean scores on the attention subscales were 52.5 on the YSR (SD 3.6; range 50–60) and 53.8 on the CBCL (SD 4.2; range 50–62), and on the ADHD subscales 53.0 on the YSR (SD 4.1, range 50–67) and 53.4 on the CBCL (SD 4.0, range 50–63). Participating adults filled out the Adult Self Report form [40]. Mean score on the attention scale was 52.6 (SD 3.5, range 50–59) and on the ADHD scale 52.8 (SD 4.2, range 50–63). None of the subjects in the analysis scored within the clinical range on the ADHD or attention subscales. Furthermore, self-reports indicated that all subjects were free of other neurological or somatic health problems.

To index IQ, subjects in the Adolescent group were administered the Vocabulary and Block Design subtests of the Wechsler Intelligence Scale for Children [41]. Subjects in the Adult group were administered the same subtests of the Wechsler Adult Intelligence Scale [36]. Mean reliability and validity of this estimated IQ-score compared to the complete IQ-test has been reported to be .9 for both scales [42], [43]. The mean IQ-score was 95.0 (SD 8.5) in the Adolescent group and 99.7 (SD 11.6) in the Adult group. IQ-scores did not significantly differ between groups (*F*(1,38) = 2.1, *p* = .154, *η_p_^2^* = .056).

### Procedure

The experimental session lasted 2.5–3 hours. The session started with three tests from the WISC-III [41] and WAIS-III [36]; the Block Design test, Vocabulary test and the Digit Span test. The latter (Digit Span Forward and Backward) was performed by the participants to obtain an independent measure of WM-capacity. Subsequently, the electrodes were attached. During the experimental session all participants sat in front of a 17-inch VGA monitor with their eyes aligned to the centre of the screen at a distance of approximately 75 cm. The participants were instructed to minimize eye blinks and to refrain from making head or eye movements during task performance. The experimental session started when all tasks were practiced until a predetermined performance criterion (75% correct responses) was reached.

### Experimental Task

To measure developmental differences in WM-capacity and the efficiency of excluding irrelevant items from access to memory, a Visual Short-Term Memory task comparable to that used by Vogel and Machizawa [31] and Vogel et al. [8] was presented to the subjects.

**Figure 1:**
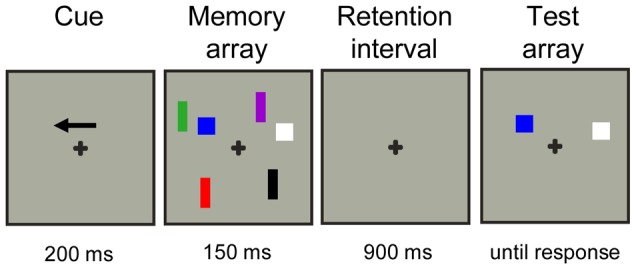
Example of distracters-present trial (T1D2) for the left hemifield.

The task consisted of bilateral stimulus displays in which colored squares (0.76°×0.76°) or rectangles (1.15°×0.57°) were presented within two 4°×7.3° rectangular regions presented 3° to the left and right from of a central fixation cross; see Figure 1). On each trial, the positions of the items were randomly distributed within upper and lower quadrants of the screen with the constraint that the distance between objects within a hemifield was at least 2° (centre to centre). The colour of squares and rectangles was randomly selected on each trial with limited replacement from a set of seven easily distinguished colours (red, blue, green, violet, yellow, black and white). A color was used only once per trial for a square or a rectangle. The number of targets and distracters was always the same in both hemifields, only location and color of the stimuli could differ between hemifields. All stimuli were presented on a grey background.

A trial started with the presentation of an arrow cue that indicated the hemifield that subjects should attend to on the following memory display. The subject's task was to remember the location and colors of the squares (T: targets) in this cued hemifield for a later test. A total of 480 trials were presented. On half of the trials the squares were accompanied by distracters (D: colored rectangles) that had to be ignored. In total, there were four different types of memory displays differing in the number of targets and distracters. Either one or three targets (squares) were presented alone (T1D0 or T3D0; memory load of 1 or 3 items) or were accompanied by two distracters (T1D2 or T3D2). The T3D2 condition was included to be able to determine whether capacity limits were reached. All memory displays were followed by a test display 900 ms later in which one colored square was presented at one of the locations in the memory display within the upper or lower quadrant (to both hemifields). The subjects had to press a left button with the left index finger when the test stimulus shown at this location had the same color as that in the previous memory display (50% of all trials) or press right with the right index finger when it was different. A new memory display followed 500–700 ms after a response was given. See Figure 1 for an example of a complete trial of the T1D2 condition with exact timing parameters.

The behavioral measures derived from the VSWM change detection task in T1D0, T1D2 and T3D0 were: 1) reaction times for correct detections (RT), 2) percentage correct responses (% Hits), and 3) *K* scores. Also an “Unnecessary Storage" measure was computed by subtracting *K*-T1D2 from *K*-T1D0.

Only reaction times to correct responses that fell within a response window from 250–4000 ms after the memory probe were included in the analysis (1.4% of responses were excluded from the analysis due to RTs that were either too fast (1.1%) or too slow (0.3%)). Cowan's memory capacity measure *K* in T1D0 and T3D0 conditions was computed with a standard formula [5]: *K* = (H+CR−1) N, in which H is the hit rate, and CR are the correct rejections in an array with N items. To derive a behavioral measure of filtering efficiency, following a study by Lee et al. [34] we also computed *K* in the distracter condition (T1D2) by filling in 1 for N since there was 1 target item; if distracters are perfectly filtered out *K* will be 1, in case of imperfect filtering *K* will be lower than 1. This *K-*T1D2 measure was subtracted from the *K*-T1D0 to obtain an “Unnecessary Storage" measure.

### Electrophysiological Recording and Analysis

For measurement of the EEG, an elastic cap (Easycap) containing 60 Ag/AgCl electrodes was used. The montage included 7 midline sites (Fpz, Fz, FCz, Cz, CPz, Pz, Oz), and 52 lateralized sites (Fp1, Fp2, AF7, AF3, AF4, AF8, F7, F5, F3, F1, F2, F4, F6, F8, FT7, FC5, FC3, FC1, FC2, FC4, FC6, FT8, T7, C5, C3, C1, C2, C4, C6, T8, TP7, CP5, CP3, CP1, CP2, CP4, CP6, TP8, P7, P5, P3, P1, P2, P4, P6, P8, PO7, PO3, PO4, PO8, O1, O2), and the right mastoid A2. During measurement all electrodes were referenced to the left mastoid (A1) and one of the electrodes in the cap (AFz) was used as ground. Offline, EEG data were re-referenced to the average of the right and left mastoids. Blinks, vertical and horizontal eye-movements were measured by bipolar electrodes placed above and below the left eye and at the outer canthi of both eyes. All electrode impedances were kept below 10 kΩ, with the exception of the reference and ground electrodes, which were held below 5 kΩ. Signal acquisition was accomplished using Brainamp amplifiers and Brain Vision Recorder software (version 1.10). EEG and EOG signals were continuously sampled at 250 Hz with a high-pass filter of 0.05 Hz and a low-pass filter of 30 Hz.

ERP analysis was done in Neuroscan 4.3.1. The continuous EEG was divided into 480 epochs of 1250 ms, from 200 ms prestimulus to 1050 ms poststimulus, all aligned to a baseline from −200 to 0 ms preceding the memory array. First, vertical (blinks) and horizontal electro-oculogram (VEOG and HEOG) artifacts were removed from the data by applying an eye-movement correction algorithm [44]. For the computation of regression coefficients between VEOG and the EEG-signals at the different electrodes, adequate eye blinks were manually selected and transmission coefficients were computed on the basis of these selected trials. In a similar way, by manually selecting horizontal eye movements separately for right- and left cued displays for each individual, regression coefficients between HEOG and the EEG-signals at the different electrodes were computed. All transmission coefficients were carefully checked to have strengths, signs and topographies congruent with expected patterns for vertical and horizontal movements before they were applied to remove eye blinks and horizontal eye movements from the EEG through the Semlitsch et al. procedure. After EOG-artifact removal, epochs still containing artifacts exceeding ±75 µV were rejected from the database. The above procedure should have removed all HEOG activity from the EEG signal. Furthermore, there is relatively little transfer of HEOG signals on parietal-occipital electrodes (for transmission coefficients see [45]). However, to exclude the possibility that residual HEOG activity that was not removed by the correction procedure had influenced CDA condition effects, we performed an extra check. We computed correlations between condition effects at HEOG and parietal-occipital electrodes in the 300–550 ms window. These correlations were all non-significant (T1D0-T3D0 effect: r(38) = .01, *p* = .97; T1D0-T1D2 effect: r(38) = .18, *p* = .28; T1D2-T3D0 effect: r(38) = −.29, *p* = .08), confirming that our CDA results cannot be explained by ocular artifacts. Next, average ERPs were computed separately for each subject in three different task conditions: (1) one target square only (T1D0; where T = number of targets and D = number of distracters), (2) one target square plus two distracter rectangles (T1D2), (3) three target squares only (T3D0). The fourth condition (T3D2) was not included in the analyses since the number of shapes (five) in this condition by far exceeded the maximum WM-capacity of about only 2 items in our adolescents and adult subjects (see T3D0-K data below), so that this condition does not provide any additional information about filtering or capacity differences between the groups. In the averaging procedure, only trials with correct responses were included. There was a maximum number of 120 trials in each task condition. Across conditions, the mean number of artifact-free EEG epochs contained in the single-subject averages was 101.29 trials (SD 15.98) in the adult group and 74.33 trials (SD 23.70) in the adolescent group.

We computed contralateral waveforms by averaging the activity recorded at right hemisphere electrode sites when subjects were cued to remember the left side of the memory array with the activity recorded from the left hemisphere electrode sites when they were cued to remember the right side. CDA was measured at posterior parietal and lateral occipital electrode sites (P1/2, P3/4, P5/6, P7/8, PO3/4, PO7/8, O1/2) as the difference in mean amplitude between the ipsilateral and contralateral waveforms. The activity from the different electrode pairs was averaged to obtain the CDA used in the analysis. This calculation method and choice of electrodes was exactly similar to that used in the original CDA studies [31] and [8]. Consistent with previous research in different labs [8], [31], [35], [46], the onset of the contralateral delay activity was around 300 ms after presentation of the memory array. In our adult data, a decrease in CDA amplitude in T1D2 and T3D0 conditions was seen in the later part of the retention interval (from about 550 ms), as has also been observed in other studies when subjects had to maintain relatively low WM loads around two items [31], [47], [48], which was close to the maximum capacity in our adults, as is indicated by Cowan's *K* measure (see Table 1). To be able to make a comparison between encoding/maintenance in both groups a measurement window from 300 to 550 ms after the onset of the memory array was selected for the analysis; comparable scoring windows for the contralateral activity in adults were used in studies by Brisson and Jolicoeur and Robitaille et al. [48], [49].

**Table 1:** Group means (and SD's) of Forward, Backward and Standardized Digit span scores (WAIS III), and WM-capacity *K* of the VSWM change detection task in adolescents and adults.

	Digit span (verbal)			WM-capacity *K* (visuospatial)		
	Forward	Backward	Standardized	T1D0	T1D2	T3D0
**Adolescents**	8.2 (1.6) *	5.4 (2.0) ***	8.8 (2.8) ***	.80 (.14)	.65 (.18) **	1.75 (.51) *
	Range:	Range:	Range:	Range:	Range:	Range:
	5–13	2–11	3–17	.42–.98	.33–.93	.78–2.50
**Adults**	10.1 (2.3)	8.4 (2.6)	12.5 (3.6)	.90 (.14)	.83 (.16)	2.16 (.48)
	Range:	Range:	Range:	Range:	Range:	Range:
	6–14	5–14	7–19	.43–.99	.39–.98	1.20–2.83

NB: Stars indicate significant group differences (*P* values after Bonferroni correction),

* *p*<.05;

** *p*<.01;

*** *p*<.005.

T1D0 = one item, no distracters; T1D2 = one item, two distracters; T3D0 = three items, no distracters.

Standard deviations between brackets.

### Statistical Analysis

#### Behavioral measures

Potential differences in verbal and VSWM-capacity between the two Age groups were tested with Bonferroni-corrected independent samples t-tests for Backward, Forward and Standardized Digit Span tests (verbal WM) and *K* in T1D0, T1D2 and T3D0 conditions (VSWM). To test for group differences in WM-access filtering efficiency two planned mixed ANOVA analyses with Trial-type (either T1D0 vs. T1D2 or T1D2 vs. T3D0) and Age as factors were performed on change detection reaction time (RT) and accuracy (% correct). With worse filtering efficiency T1D0-T1D2 RT differences will increase and T1D2-T3D0 RT differences will decrease due to longer search times in T1D2 (due to unnecessary storage of distracters). In the same way T1D0-T1D2 accuracy differences will increase and T1D2-T3D0 accuracy differences will decrease. An independent t-test was performed to test whether adolescents and adults had different Unnecessary Storage scores (difference score between K-T1D0 and K-T1D2).

#### ERP measures

For the parietal/occipital CDA window (300–550 ms), mean amplitudes were compared across conditions by three planned mixed ANOVA's, with a within-subject factor Trial-type with two levels (T1D0 & T3D0 to examine load effects, or T1D0 & T1D2 or T1D2 & T3D0 to examine distracter effects) and a between-subjects factor Age (adolescents, adults). In case of significant Age×Trial-type interactions, post-hoc tests were performed for the separate groups and for the separate conditions.

#### Correlations

To examine whether filtering efficiency was related to WM-capacity, correlations between WM-capacity measures and Unnecessary Storage were calculated using Pearson correlation coefficients. Furthermore, to examine correlations between performance and electrophysiological measures of distracter interference, correlations between behavioral differences in T1D0 and T1D2 and CDA amplitude differences in T1D0 and T1D2 for the CDA window were calculated.

## Results

### Behavioral Results

#### Developmental differences in verbal (Digit Span) and visuospatial span (Cowan's *K*)

**Figure 2:**
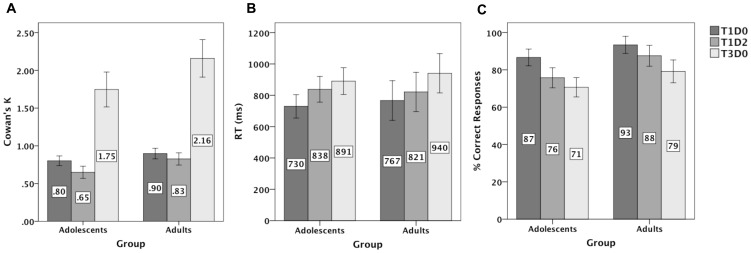
Behavioral data from the VSWM change detection task. Bar graphs of *(A)* Cowan's *K*, *(B)* average reaction times (in ms), and *(C)* percentage of correct responses for adolescents and adults in T1D0 (one target), T1D2 (one target, two distracters) and T3D0 (three targets) conditions. Error bars indicate 95% confidence intervals.

Forward, Backward and Standardized spans were collected for both groups to obtain verbal WM span measures and visuospatial span was measured by computing *K* in T1D0 and T3D0 conditions of the present delayed-WM task. For means and standard deviations of scores in the Digit Span task see Table 1. For *K* scores (and SD's) in T1D0, T3D0 and T1D2 conditions see Table 1 and Figure 2A, Panel A. Bonferroni-corrected independent t-tests showed significantly lower Forward, Backward and Standardized Digit Span scores in adolescents than adults (Forward Digit Span: *t*(36) = −2.99, *p*<.05, Cohen's effect size *d* = −0.997; Backward Digit Span: *t*(36) = −3.94, *p*<.005, *d* = −1.31; Standardized Digit Span: *t*(36) = −3.52, *p*<.005, *d* = −1.17). Bonferroni-corrected independent t-tests for *K* showed a trend towards lower *K* scores in adolescents than adults in T1D0 (*t*(36) = −2.1, *p* = .13, *d* = −0.70), and significantly lower *K* scores in adolescents than adults in T3D0 (*t*(36) = −2.5, *p*<.05, *d* = −0.83) and T1D2 (*t*(36) = −3.2, *p*<.01, *d* = −1.07) conditions.

#### Effect of distracters on the speed (RT) of change detection performance

For means of reaction times (and 95% confidence intervals) for adolescents and adults, see Figure 2, panel B.

The ANOVA for the T1D0 vs. T1D2 comparison showed a significant main effect of Trial-type (*F*(1,36) = 26.1, *p* = .0001, *η_p_^2^* = .420) and a trend-significant Age×Trial-type interaction (*F*(1,36) = 2.8, *p* = .10, *η_p_^2^* = .073) in the expected direction; adolescents showed a steeper increase than adults in the time needed to make an accurate memory decision for 1 item when it was accompanied by distracters compared to when it was not (RT-T1D0-T1D2 Trial-type effect in adolescents: *F*(1,20) = 24.8, *p*<.00001, *η_p_^2^* = .553 and in adults: (*F*(1,16) = 5.6, *p*<.05, *η_p_^2^* = .259); see Figure 2, Panel B.

For the T1D2 vs. T3D0 comparison a significant Age×Trial-type interaction (*F*(1,36) = 5.5, *p*<.05, *η_p_^2^* = .133) in the expected direction was found; adults showed a larger increase in RT from T1D2 to T3D0 than adolescents (119 ms and 53 ms respectively) (RT-T1D2-T3D0 Trial-type effect in adolescents (*F*(1,20) = 6.2, *p*<.05, *η_p_^2^* = .236) and in adults (*F*(1,16) = 43.5, *p*<.00001, *η_p_^2^* = .736)); see Figure 2, Panel B.

#### Effects of distracters on the accuracy of change detection performance

Memory accuracy (proportion of correct responses) for both groups is shown in Figure 2, panel C.

A significant Age×Trial-type interaction in the expected direction was found for the T1D0 vs. T1D2 comparison (*F*(1,36) = 4.2, *p*<.05, *η_p_^2^* = .104), due to adolescents showing a steeper decline in memory accuracy for a WM-load of 1 item when it was accompanied by distracters than adults (T1D0-T1D2 effect adolescents: *F*(1,20) = 30.7, *p*<.0001, *η_p_^2^* = .605; T1D0-T1D2 effect adults: *F*(1,16) = 20.8, *p*<.001, *η_p_^2^* = .566).

The T1D2 vs. T3D0 comparison did not yield a significant interaction between Age and Trial-type, but resulted in a main effect of Trial-type (*F*(1,36) = 32.6, *p*<.00001, *η_p_^2^* = .476) caused by reduced accuracy when holding 3 items in memory compared to holding 1 item in the presence of distracters. Furthermore, a main group effect (*F*(1,36) = 8.1, *p*<.01, *η_p_^2^* = .184) showed overall higher accuracy in adults than adolescents across the T1D2 and T3D0 conditions. The Unnecessary Storage measure derived from *K* scores in T1D2 and T1D0 conditions (see Methods page 11) was significantly higher in adolescents (.15) than in adults (.07) (*t*(36) = 2.3, *p*<.05, *Cohen's d* = 0.77), confirming higher storage of distracters in the former group.

### ERP results

**Figure 3:**
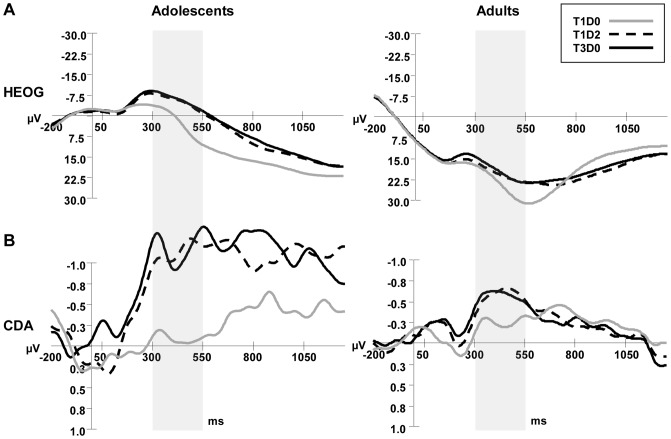
Average ERP activity during the VSWM change detection task. *(A)* HEOG activity ((HEOG left visual field trials*-1+HEOG right visual field trials)/2) and *(B)* CDA activity (computed by subtracting ipisilateral from contralateral activity), after smoothing with a 6 Hz low-pass filter, time-locked to the memory array and averaged across occipital and posterior parietal electrode sites for adolescents and adults, in conditions T1D0, T1D2 and T3D0. The analyzed window (300–550 ms) is indicated by a grey rectangle.

Grand ERP averages of CDA (VEOG/HEOG corrected) at occipital and parietal sites (P1/2, P3/4, P5/6, P7/8, PO3/4, PO7/8, O1/2) and average HEOG in the Adolescent and Adult group for the three task conditions are depicted in Figure 3. Mean amplitudes and SDs of the CDA amplitude in the predefined 300–550 time window per task condition and age group are shown in Table 2.

**Table 2:** Mean CDA amplitudes (in µV, standard deviations between brackets) for adolescents and adults in the VSWM change detection task, averaged over lateral parietal and occipital electrode sites.

		T1D0	T1D2	T3D0
**CDA (300–550 ms)**	**Adolescents**	−0.14 (1.62)	−1.27 (1.40)	−1.18 (1.55)
	**Adults**	−0.15 (0.45)	−0.50 (0.49)	−0.53 (0.40)

NB: T1D0 = one item, no distracters; T1D2 = one item, two distracters; T3D0 = three items, no distracters.

#### Effects of load on CDA

Mean amplitudes of parietal-occipital CDA in T1D0 and T3D0 conditions were entered into a repeated measures analysis of variance to test for age differences in WM-load-related CDA increases. A significant Age×Trial-type interaction was found for the T1D0 and T3D0 comparison (*F*(1,36) = 4.6, *p*<.05, *η_p_^2^* = .112), indicating that the load related CDA amplitude increase from 1 to 3 items was larger in adolescents than adults (T1D0-T3D0 effect in adults: *F*(1,16) = 24.6, *p*<.00001, *η_p_^2^* = .606; T1D0-T3D0 effect in adolescents: *F*(1,20) = 14.9, *p*<.005, *η_p_^2^* = .428). Post-hoc univariate ANOVA tests showed that there was a trend for adolescents to have higher T3D0 amplitudes than adults (Age effect F (1,36) = 2.9, *p*<.10, *η_p_^2^* = .074), whereas T1D0 amplitudes were not different between the groups (*F*(1,36) = .0, *p* = .98, *η_p_^2^* = .000).

#### Effects of distracters on CDA

The comparison between T1D0 and T1D2 also showed a significant interaction between Age and Trial-type (*F*(1,36) = 6.0, *p*<.05, *η_p_^2^* = .143), reflecting the expected larger CDA amplitude increase from T1D0 to T1D2 in adolescents (*F*(1,20) = 17.7, *p*<.00001, *η_p_^2^* = .469) than in adults (*F*(1,16) = 10.5, *p*<.01, *η_p_^2^* = .396). Follow-up univariate ANOVA tests showed that the interaction was caused by significantly higher T1D2 CDA amplitudes in adolescents than adults (*F*(1,36) = 4.6, *p*<.05, *η_p_^2^* = .113), whereas this was not the case in T1D0 as shown in the previous paragraph. This presence of a group difference in CDA amplitude only in the distracter-present condition is evidence for increased distracter processing in adolescents. The absence of Trial-type or Trial-Type×Age effects for the T1D2-T3D0 CDA comparison indicates CDA overlap between these conditions in both groups as can be seen in Figure 3.

**Figure 4:**
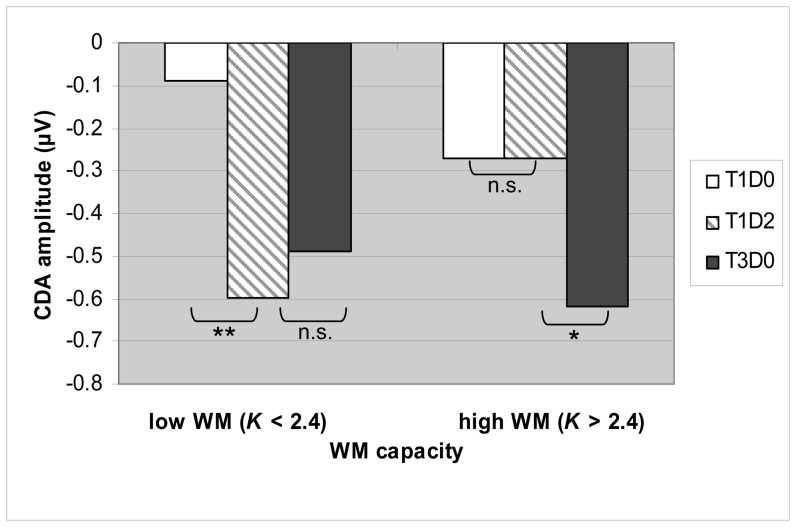
CDA amplitudes for adults with high and low *K*-scores. Mean amplitudes between 300 and 550 ms in T1D0, T1D2 and T3D0 conditions in adults with high (N = 5) and low (N = 12) working memory capacity (WMC), determined by a *K*-T3D0 score larger or smaller than 2.4. * *p* = .087, one-tailed, ** *p*<.001, one-tailed, n.s. = non-significant.

In the initial CDA-WM-filtering study in Nature by Vogel et al. [8], adults with high WM-capacity showed a pattern of smaller T1D0-T1D2 CDA differences but higher T3D0 -T1D2 CDA differences during encoding/maintenance than adults with low-capacity because of perfect filtering of distracters. Our adults did show smaller T1D2-T1D0 amplitude differences than adolescents, but did not show smaller T1D2 than T3D0 CDA amplitudes. Considering the CDA literature that only adults with high WM-capacity (high *K* scores) show T3D0-T1D2 differences [8], the T1D2 and T3D0 CDA overlap in our adults can be explained by the fact that the majority of our adults tended towards low capacity as becomes evident from their low mean *K* score of about 2 items. Since CDA amplitude reaches its maximum amplitude at maximum capacity, the overlap between T3D0-T1D2 CDA is likely caused by a “ceiling" effect in T3D0 in our adults because CDA reached its maximum with storage of two items. To support such an explanation by data, we created high (*K* T3D0 score ≥2.4) and low (*K* T3D0 score <2.4) WM-capacity adult groups and performed an ANOVA to see if we could also find the patterns reported in Vogel's study for subjects with relatively high and low WM-capacity. The criterion of >2.4 was used to select subjects with a *K*-score that was clearly and significantly higher than the total group's mean K-score of 2.16. This high/low split resulted in a group of 5 adults with high WM-capacity (mean *K* scores of 2.66, SD .14) and a group of 12 low WM-capacity adults (mean *K* score of 1.95, SD .42); this difference in *K* was highly significant (*t*(15) = −5.2, *p*<.001, *d* = −2.69). Congruent with CDA patterns reported in Vogel et al. the repeated measures ANOVA analyses yielded a significant WM-capacity×Trial-type interaction for the T1D2-T3D0 CDA amplitude comparison (*F*(1,15) = 5.9, *p*<.05, *η_p_^2^* = .282), caused by a trend significant Trial-type effect of higher T3D0 than T1D2 amplitudes in high-span subjects (*t*(4) = −1.66, *p* = .087, one-tailed, *d* = −1.66) and the absence of a T1D2-T3D0 CDA difference in low-span subjects (*t*(11) = 1.24, *p* = .121, one tailed, *d* = 0.75). Also congruent with findings by Vogel et al., a significant WM-capacity×Trial-type interaction (*F*(1,15) = 5.6, *p*<.05, *η_p_^2^* = .278) was found for the T1D2-T1D0 CDA amplitude comparison, this time caused by a significant T1D2 (vs. T1D0) CDA increase only in the low-capacity group (*t*(11) = −4.75, *p*<.001, one-tailed, *d* = −2.86), but not in the high-capacity group (*t*(4) = 0.02, *p* = .494, one-tailed, *d* = 0.002); see Figure 4 for mean amplitudes in T1D0, T1D2 and T3D0 in both groups.

### Correlations between performance and electrophysiological CDA measures

**Figure 5:**
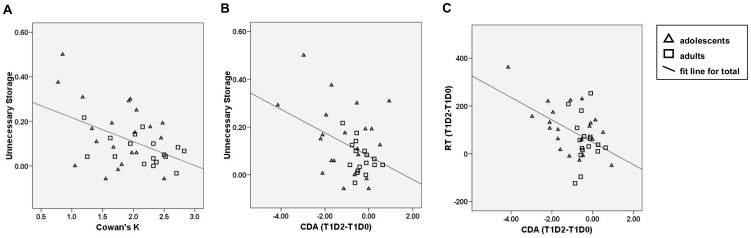
Scatterplots of significant correlations between behavioral and CDA measures. (*A*) Correlation between *K*-T3D0 and Unnecessary Storage (*K*-T1D0 minus *K*-T1D2) for adolescents (triangles) and adults (squares). *(B&C)* Correlation between distracter related parietal-occipital CDA effects (CDA-T1D2 minus CDA-T1D0) and Unnecessary Storage (*K*-T1D0-*K*-T1D2; panel *B*) or RT distracter effects (RT-T1D2 minus RT-T1D0; panel *C*). Negative CDA effects are observed when T1D2 CDA amplitude is larger than T1D0 CDA amplitude. Larger negative CDA (T1D2-T1D0) values reflect larger CDA increases when distracters are present. Larger positive distracter-related RT values reflect larger RT increases when distracters are present.

Correlations were computed between performance and CDA measures of WM-capacity and filtering efficiency. Figure 5 depicts the significant correlations.

*K* (T3D0) was significantly correlated (r(38) = −.46, *p* = .003) with the Unnecessary Storage measure (*K*-T1D0 minus *K*-T1D2); subjects with higher WM-capacity had lower Unnecessary Storage scores (see Figure 5A).

The Unnecessary Storage measure also correlated negatively with distracter-related CDA effects (T1D2 amplitude - T1D0 amplitude) from 300–550 ms (r(38) = −.41, *p* = .011) (see Figure 5B). Similar negative correlations were found between distracter-related RT increases (RT-T1D2 minus RT- T1D0) and distracter-related CDA increases in the same conditions (r(38) = −.48, *p* = .002; see Figure 5C). These correlations indicate that individuals with larger distracter interference effects on accuracy (reflected by the Unnecessary Storage measure) and speed of memory performance also had larger distracter-related CDA amplitude increases and individuals with low distracter interference effects on performance showed small distracter-related CDA amplitude increases. This is evidence for a functional relation between performance and CDA measures of distracter interference.

## Discussion

Several behavioral studies have shown that adolescents have still immature WM performance, mainly in tasks requiring high levels of executive control [19], [20], [21]. This raises the question of whether this worse performance than adults is caused by still immature WM processing capacity during the encoding of items for maintenance in WM or whether other processes like memory comparison or response decision times also play a role. Furthermore, it is not yet known to what extent inefficient WM-filtering mechanisms play a role in limiting working memory capacity at this age by using up capacity by inefficient filtering of goal-irrelevant information. To answer these questions, a delayed visuospatial WM (VSWM) change detection task with manipulations of WM-load and distraction [13] was administered to adolescents and adults in the current study. Furthermore, in addition to change detection performance, contralateral delay activity (CDA) was measured, as a time-sensitive neural correlate of the number of targets and distracters that subjects encoded and maintained in WM during the delay interval of the task.

### Development of VSWM-capacity throughout adolescence

The behavioral results showed that adolescents indeed had lower verbal WM-capacity than adults as measured by the Digit Span task and lower visuospatial WM-capacity as measured by Cowan's *K* in the VSWM change detection task. Adolescents made significantly more memory errors (29% vs. 21%) and had lower *K* scores than adults (1.75 vs. 2.16) when three target items had to be maintained in VSWM. The present study shows that adolescents have immature capacity in a relatively simple VSWM change detection task in which they have to store a maximum of three items. Our findings seem to be in contrast with findings by other studies using VSWM change detection tasks that reported mature VSWM-capacity already at age 10–12 [10], [16], [50]. This discrepancy can however be explained by differences in task parameters. In our study memory array presentation duration and maintenance delays were shorter than in the other developmental studies. Longer delays might have enabled the use of verbal coding strategies that help to improve visual memory in older children. It is known that older children are prone to use verbal strategies to enhance visual memory [51]. Vogel, Woodman, & Luck [52] showed that with the currently used presentation duration of 150 milliseconds and total maintenance delay of 1050 ms, the use of verbal coding strategies is highly unlikely. Furthermore, selective attention demands were probably larger in our task because of the bilateral stimulus displays that also required the subjects to ignore the irrelevant items presented in the unattended visual field.

If the lower VSWM-capacity in adolescents is to be explained by lower storage capacity, this was expected to become visible as smaller load-related CDA increases in adolescents than adults. Both adults and adolescents showed the normally reported CDA amplitude increase with load from 300–550 ms after presentation of the memory display, when encoding and maintenance is taking place [31], [32], [34], [35], [53]. This main Trial-type effect was however qualified by a significant Trial-type×Age interaction, showing that whereas CDA amplitudes did not at all differ between adolescents and adults when encoding/maintaining a low 1-item load in WM, adolescents tended to have a higher CDA amplitude than adults when the load increased to 3 items. Although this effect was clearly visible in the ERP plots, it was only trend significant, probably due to larger variability of T3D0 CDA amplitudes in adolescents. This trend towards *higher* CDA amplitude increases with load in adolescents is against expectations and does not seem to be in line with an interpretation of CDA amplitude reflecting the number of items stored since the *K* measure indicated that adolescents stored less items in the T3D0 condition than adults. Instead, the higher CDA increase with load in adolescents might be interpreted as the recruitment of more processing resources (possibly spatial attention) needed for storage of relatively high loads. In the only other developmental CDA study that we now of that included younger children, Sander, Werkle-Bergner, & Lindenberger [54] reported an absence of load-related CDA responses in younger, 10–12 year-old children when presentation times were comparable to that in the present study (100 ms). The fact that we did find significant CDA load effects in our older adolescent group even with short presentation times suggests that the neurocognitive mechanisms underlying WM-capacity undergo important development between late childhood and late adolescence.

Summarizing the developmental load-related behavioural and CDA results; adolescents had lower verbal and visuospatial WM span than adults and whereas CDA data showed significant load-related CDA responses in both adolescents and adults, CDA amplitudes tended to be higher in adolescents than adults only in the high-load (3 item) condition. This finding could point to the need for recruitment of more parietal resources for WM encoding/maintenance by adolescents when relatively high visuospatial loads (e.g. 3 items) have to be processed and is congruent with the late development of frontal-parietal networks underlying VSWM performance [22], [55].

### Development of filtering efficiency in VSWM in adolescence

The hypothesis that adolescents' WM performance in a visuospatial change detection task would suffer more from the presence of distracters in the memory array than that of adults was confirmed by accuracy, *K* and reaction time data. The presence of distracters in the memory array (T1D2) led to a larger drop in memory accuracy for the target in adolescents than adults. This was shown by a significant Age×Trial-type interaction for accuracy data (T1D0 vs. T1D2 comparison) and a significantly larger Unnecessary Storage score in adolescents than adults. This Unnecessary Storage score was derived by subtracting *K* in T1D2 (1 target, 2 distracters) from *K* in T1D0 (no distracters); in case of storage of distracters the Unnecessary Storage score is higher than 0 [34], which was the case in both groups, but more so in adolescents. Unnecessary Storage was also significantly correlated with *K*, showing that subjects with higher WM-capacity showed less interference from distracters on their WM performance. Besides these effects on accuracy, Age×Trial-type interactions for reaction time (RT) showed the predicted larger T1D0-T1D2 (at two-tailed *p* = .10 level of significance) and smaller T1D2-T3D0 reaction time differences in adolescents than adults. These reaction time effects are caused by the larger slowing of change detection response times by the presence of distracters in adolescents than adults. Cowan and Morey [9] suggested that especially low-WM-capacity individuals might use the strategy to store and maintain the entire stimulus display and only decide afterwards whether any changes are visible in the test array. This strategy would result in relatively longer reaction times in distracter-conditions, which were observed for adolescents (versus adults) in the present study.

The CDA was measured in addition to the above discussed performance measures to study the online storage of distracters during the delay interval of the task before a response was generated. We hypothesized on the basis of the change detection-CDA literature that larger WM-distracter interference in adolescents would be due to more problems with blocking distracter items from encoding and maintenance in WM. If so, this would show itself in the CDA data by larger T1D2 amplitude increases, relative to that in T1D0, in adolescents than in adults. Significant Age×Trial-type (T1D0-T1D2) CDA interaction effects during encoding/maintenance confirmed significantly higher T1D2-CDA amplitudes in adolescents than adults, whereas T1D0 amplitudes were comparable between both age groups. In line with the change detection-CDA literature these larger distracter-related CDA increases in adolescents signify higher encoding and maintenance of distracter items in WM [8], [33], [34]. This was further confirmed by the positive correlation that was found between distracter-related performance and CDA effects; subjects with higher parietal-occipital CDA T1D2-T1D0 amplitude increases also had higher Unnecessary Storage performance scores and showed stronger distracter interference effects on reaction time.

It has to be noted that, in both adolescents and adults T1D2 amplitude overlapped with T3D0 amplitude whereas in case of more efficient filtering in adults one might expect that, besides the smaller T1D0-T1D2 difference that we found, adults would also show higher T3D0 than T1D2 CDA amplitude, as has been reported in adults with high WM-capacity [8]. However, our adult subjects on average had a low WM-capacity of about only two items, as shown by their *K* score in the T3D0 condition. Since CDA amplitude is known to reach its maximum amplitude with an individual's maximum capacity [31], it is assumable that the T3D0-T1D2 overlap in our adult group is caused by the fact that the mean CDA amplitude already reached its maximum with storage of two items and could not further increase (e.g. reached a “ceiling”) in the T3D0 condition. To support this by data we performed an analysis in which we formed high and low-span adult groups based on their *K* scores and investigated whether adults with relatively high WM-capacity (mean *K* score of 2.66) would show significantly higher T3D0 than T1D2 CDA amplitudes (and overlapping T1D0-T1D2 CDA) as found in high-span subjects in the initial [8] study, whereas adults with relatively low *K* scores (mean 1.95) would not. Significant Trial-type×WM-capacity interactions for both the T1D2-T1D0 and the T1D2-T3D0 CDA comparisons confirmed the patterns earlier reported by Vogel et al. [8]. Follow-up post-hoc tests confirmed the expected T1D0<T1D2 = T3D0 CDA pattern in low capacity adults and a T1D0 = T1D2<T3D0 CDA pattern in high capacity adults (although the T1D2-T3D0 difference in the high capacity group was only marginally significant due to the small sample size). So this might explain why in the present study higher distracter encoding and maintenance in adolescents than adults (including all subjects) was only significant when comparing T1D0 and T1D2 CDA amplitudes.

The reason for the lower WM-capacity and imperfect filtering performance in our adults compared to that in other studies is explained by the fact that we selected adults with moderate education levels from the normal population with the purpose of matching them on education levels (and thereby IQ) with adolescents. This resulted in an adult sample with relatively lower IQ scores (99) than that in other studies that mostly included university students. This did lead to the absence of IQ differences between our adolescent and adult groups, which is very important in cognitive developmental research, since if groups are not matched on IQ it is impossible to exclude that WM differences are due to general IQ differences. This is especially important in WM research since WM-capacity is known to be related to fluid intelligence. Also in our study IQ was positively correlated with WM-capacity (the latter measured by WAIS Digit Span and *K* in the VSWM task), IQ explained 35% of the variance in *K*. Interestingly, Fukuda et al. [4] recently reported data that led to the conclusion that this IQ-WM-span relationship is mediated by the number of representations that can be simultaneously maintained in WM, rather than by the precision of those representations.

Summarizing the developmental filtering efficiency results, our performance measures confirmed our hypothesis of worse WM filtering (higher distracter interference) in adolescents. CDA data also partly showed that this was due to higher distracter processing during encoding/maintenance, since adolescents' CDA increased more than that of adults when holding 1 item in WM in the presence of distracters, whereas there was no difference in CDA amplitude between the groups in the 1-item distracter absent condition.

A possible limitation of this study might be that a wide age range was used for the adolescent group, in which developmental changes in VSWM capacity and filtering efficiency could have taken place. A comparison between young (12–14 years old, N = 12) and old adolescents (15–16 years old, N = 9) however showed no significant differences in behavioral measures (digit span, Cowan's *K*, Unnecessary Storage and RT increase and % Hits decrease with load and distracters) or electrophysiological measures (CDA increases with load or distracters) of WM capacity and filtering efficiency. Twelve-year-old adolescents performed similar to 16-year-old adolescents in the present change detection task, and it is therefore unlikely that developmental changes in VSWM capacity or filtering efficiency within the adolescent group have influenced the present results.

### Conclusion

In conclusion, the present study showed that adolescents performed worse than adults in a VSWM change detection task. Performance data showed that adolescents had lower WM capacity than adults (evident from lower digit span and lower Cowan's *K* scores in the visual change detection task). Against expectations, the CDA increase with load was larger (instead of smaller) in adolescents than in adults. This might mean that in adolescents the CDA amplitude reflects something else than the number of items stored, perhaps the allocation of processing resources such as spatial attention. Furthermore, adolescents were less efficient than adults in blocking distracter items from processing in WM (reflected by higher CDA amplitude increases in adolescents when distracters are present in the memory display vs. when they were not).
